# Recent advances in surgical management of urinary incontinence

**DOI:** 10.12688/f1000research.16356.1

**Published:** 2019-07-31

**Authors:** Alison Downey, Richard D. Inman

**Affiliations:** 1 Department of Urology, Royal Hallamshire Hospital, Glossop Road, Sheffield, S10 2JF, UK

**Keywords:** Surgery, Urinary Incontinence, Bladder, stress incontinence, urgency incontinence

## Abstract

There have been several recent developments in surgical treatments for male and female incontinence. This article reviews the current options for treatments of urge and stress incontinence in men and women. Treatments for urge incontinence discussed include intradetrusor onabotulinum toxin A, sacral neuromodulation and percutaneous tibial nerve stimulation. For stress incontinence, suburethral mesh, bulking agents, autologous slings, colposuspension, male slings and artificial urinary sphincters are assessed.

## Introduction

Urinary incontinence is defined by the International Continence Society as any involuntary leak of urine ^1^. Classification of urinary incontinence includes stress incontinence, urgency incontinence, mixed incontinence and continuous incontinence (for example, due to vesicovaginal fistula). This review concentrates on the surgical management of urgency and stress incontinence.

## Urge urinary incontinence

Urge urinary incontinence (UUI) is defined as involuntary leakage of urine accompanied or immediately preceded by urgency and is part of the symptom complex of overactive bladder syndrome (OAB) ^1^. A systematic review of global prevalence and economic burden of UUI estimated a prevalence of between 1.85 and 30.5% in Europe and an annual cost of $7 billion in Canada, German, Italy, Spain, Sweden and the UK ^2^.

Non-surgical management of OAB consists of a stepwise approach. First-line options include lifestyle modifications such as bladder drill (or timed voiding, a self-help regimen designed to increase the interval between voids by using a graduated approach), reducing intake of caffeinated drinks, and preventative measures such as treatment of constipation and weight loss ^3– 5^.

If conservative methods are not sufficient, second-line therapies involve adding anticholinergic medications and, more recently, mirabegron, a beta-adrenergic receptor agonist approved in 2012 and recommended as a second-line treatment for UUI by the European Association of Urology (EAU), the American Association of Urology (AUA) and the National Institute for Health and Clinical Excellence (NICE) ^6, 7^.

When patients are resistant to or cannot tolerate both conservative and pharmacological treatment, surgical techniques such as intravesical onabotulinum toxin A (oBTXA), sacral neuromodulation, and posterior tibial nerve stimulation are third-line options. In those refractory to all treatments, augmentation cystoplasty or urinary diversion can be considered as a final option.

## Onabotulinum toxin A

Injection of oBTXA, a potent neurotoxin, into the bladder wall is now recommended for use in patients who have OAB with or without UUI and who have failed to respond to conservative and drug treatments. It is injected by using a flexible or rigid cystoscope into the detrusor muscle under local or general anaesthetic. The main side effects of treatment are urinary tract infection (UTI), incomplete bladder emptying and urinary retention requiring clean intermittent self-catheterisation ^8^.

Substantial high-quality data comparing oBTXA with placebo and anticholinergic medication support the use of oBTXA as a third-line treatment for patients with UUI. In 2017, in a double-blinded placebo-controlled randomised controlled trial (RCT) of nearly 500 patients, Nitti *et al*. compared 100 units of oBTXA with placebo and found that oBTXA significantly decreased the frequency of UUI episodes compared with placebo (by −2.65 versus 0.87) ^9^. Complete continence also improved in 22.9% of patients having oBTXA compared with 6.5% with placebo at 24 weeks. Side effects of oBTXA included urinary retention and UTI ( Table 1) ^9^.

**Table 1.** Comparison of onabotulinum toxin A versus placebo in treating urge urinary incontinence.

At 12 weeks	Placebo	100 units of onabotulinum toxin A	*P* value
Reduction in frequency	–0.87 (12%)	–2.65 (49%)	<0.01
Continent	6.5%	22.9%	
Urinary tract infection	9.2%	24.5%	
PVR of 200 mL or increase of 200 mL from baseline PVR	0%	8.7%	
PVR of 200 mL or greater requiring CISC	0.4%	5.8%	

Data are from Nitti *et al*. ^9^ (2017). CISC, clean intermittent self-catheterisation; PVR, post-void residual urine volume.

A 2017 systemic review comparing oBTXA with anticholinergics and mirabegron found that 100 units of oBTXA resulted in the greatest reduction in episodes of UUI and of continence (100% reduction in UUI) at 12 weeks, compared with all other licenced medication, although there was no difference in urinary frequency between oBTXA and either solifenacin 10 mg once a day or oxybutynin 10 mg controlled release once a day ^10^. However, there is currently a lack of direct head-to-head trials of oBTXA compared with anticholinergics and mirabegron.

As a result of the high-quality data available, most guidelines recommend oBTXA for refractory OAB symptoms. NICE now recommends offering oBTXA to patients with proven detrusor overactivity at an initial dose of 200 units; however, EAU guidelines recommend an initial dose of 100 units ^6, 7^. Patients should be counselled about the 5% risk of urinary retention requiring clean intermittent self-catheterisation, the requirement for repeat injections every 6 to 9 months and the increased risk of associated UTI.

## Sacral neuromodulation

Sacral neuromodulation (SNM) involves the placement of an electrode into the third sacral (S3) foramen which is connected to a generator and battery, electrically stimulating the nerve root and suppressing the reflexes responsible for involuntary detrusor contractions. Currently, sacral nerve stimulation (SNS) is recommended in those who have failed or cannot tolerate conservative and pharmacological treatment, as an alternative to oBTXA (EAU ^6^) or those unsuitable for oBTXA.

Historically, implantation of a permanent SNS generator was preceded by a trial of percutaneous electrode linked to an external stimulator for a trial of stimulation, and success was defined as more than 50% clinical improvement after 1 to 2 weeks. At a later date, a permanent tined lead and battery were implanted.

Recently, in 2015, a permanent tined electrode was inserted initially for the trial period and a permanent generator was implanted 2 weeks later without removal of the tined lead if the trial was successful ^11^. Comparison of the two implantation techniques has shown that the two-stage technique results in a higher rate of generator implantation and this is probably due to the improved stability of a permanent tined lead (50.9% versus 24.1%) ^11^.

In a 2018 systematic review of neuromodulation, Tutolo *et al*. showed that, in those with UUI, between 43 and 56% of patients were dry and 54 to 59% were improved and that the long-term results are maintained at 4 years ^12^. SNS is a safe procedure; the commonest side effects are pain at the implantation site in 15 to 42%, revision surgery in 9 to 33% and wound infections in 3 to 6%. Despite the promising results, studies of SNS have often not blinded assessors to treatments and patient allocation has been open to bias.

However, the recently reported ROSETTA (Refractory Overactive Bladder: Sacral NEuromodulation vs. BoTulinum Toxin Assessment) trial was a good-quality multi-centre open-label randomised study comparing oBTXA (200 units) with SNS in 386 women ^13^. oBTXA treatment had a small but statistically significantly increased reduction in mean number of UUI episodes (−3.9 versus −3.3) at 6 months. The oBTXA group had a higher rate of UTI (35% versus 11%) and requirement for self-catherisation (8% at 1 month and 2% at 6 months). Although there was a statistical difference in results between SNS and oBTXA, the clinical relevance is uncertain, particularly with the difference in adverse events.

In 2017, Noblett *et al*. reported the results of a prospective multi-centre study to evaluate sacral neuromodulation over a period of 12 months and found that 80% of patients underwent permanent SNM implantation following a test period ^14^. Patients with UUI had a mean reduction of 2.2 ± 2.7 leaks per day following implantation ( *P* <0.0001) ^14^. In 2014, Peeters *et al*. found that SNM was effective in the long term; the success rate was 70% after a mean follow-up of 46.88 months in patients with UUI ^15^. A summary of SNS and PTNS is provided in Table 2.

**Table 2.** Sacral nerve stimulation versus percutaneous nerve stimulation in urge urinary incontinence.

	Sacral nerve stimulation	Percutaneous nerve stimulation
Improvement in lower urinary tract symptoms	61–90%	54–79%
Failure	4–64%	40–44%
Revision surgery	9–33%	

Data are from Tutolo *et al*. ^12^ (2018).

Currently, NICE guidelines recommend offering SNM to patients if they have not responded to conservative management, including drugs, and they are unable to perform clean intermittent catheterisation (that is, they would be unsuitable for oBTXA) whereas EAU guidelines recommend offering SNM to patients who have UUI refractory to anticholinergic therapy ^6, 7^.

## Percutaneous tibial nerve stimulation

Percutaneous tibial nerve stimulation (PTNS) involves stimulating the sacral nerve plexus for 30 minutes weekly for 12 weeks by placing a needle percutaneously into the posterior tibial nerve peripherally ^16^. A 2018 systematic review of PTNS found rates of success (cure or improvement) of between 54 and 79%, depending on the definition of success ^12^. An RCT comparing PTNS with tolterodine found that 79.5% of patients with PTNS had a subjective cure or improvement in symptoms at 12 weeks compared with 54.8% of the tolterodine group, suggesting that PTNS may be a reasonable alternative to anticholinergics ^17^. MacDiarmid *et al*. followed up the patients who had responded to PTNS, further treatment being given “on demand”, and found that the effects of PTNS were sustained between 3 and 12 months ^18^. Despite encouraging data, long-term data for PTNS are sparse; most studies provide data to 12 weeks only and use varying definitions of success.

## Conclusions

Although initial results for PTNS are encouraging, high-level evidence and long-term follow-up data are lacking. It is recommended by NICE as an option only for patients who fail conservative management and do not want or cannot tolerate botulinum toxin or sacral neuromodulation following multi-disciplinary team discussion. SNS treatment for UUI symptoms in OAB is safe and effective in both the short and long term. The ROSETTA trial has shown a statistically significant reduction in UUI with use of oBTXA and SNS. Although the oBTXA group had a significantly increased effect on UUI compared to SNS, the clinical significance remains unclear, particularly as oBTXA has a different side effect profile. At present, both EAU and NICE guidelines recommend that either can be used equally for patients with refractory UUI ^6, 7^.

## Stress urinary incontinence in women

Stress urinary incontinence (SUI) is the involuntary loss of urine on effort or physical exertion, including sneezing or coughing ^1^. In women, prevalence ranges from 29 to 75%, depending on age, and two broad pathophysiological mechanisms are proposed: urethral hypermobility (weakness in the supporting mechanism of the urethra) and intrinsic sphincter deficiency (defective urethral sphincter mechanism).

Initial management of SUI includes weight reduction, pelvic floor muscle training and biofeedback ^4, 5^. Incontinence pessaries have been used in the past for women who are poor surgical candidates, as has duloxetine, a serotonin and noradrenaline reuptake inhibitor, but neither is recommended by NICE. If conservative management for SUI fails, patients may be offered surgical treatment ^7^. Surgical options for women include urethral bulking agents and bladder neck suspension procedures, including mid-urethral mesh procedures, pubovaginal slings, colposuspension, and the artificial urinary sphincter (AUS).

## Urethral bulking agents

The injection of bulking agents into the urethral submucosa is a minimally invasive treatment for SUI, improving mucosal coaptation and thus increasing urethral outlet resistance. Several agents are available for use ( Table 3) whereas others have been withdrawn because of adverse events (Zuidex, Uryz, Polytef and autologous fat). The procedure may be performed in the outpatient day case setting under local anaesthetic. Complications include urethral discomfort, overactive bladder syndrome, temporary urinary incontinence and UTIs ^22^.

**Table 3.** Periurethral bulking agents for stress incontinence.

Bulking agent	Commercial name	Current usage
Autologous fat	Autologous fat	Trial terminated (serious adverse event)
Carbon coated zirconium beads	Durasphere	Recommended by NICE
Polyacrylamide hydrogel	Bulkamid	
Calcium hydroxlapatite	Coaptite	Approved by FDA
Glutaraldehyde X linked with bovine collagen	Contigen	Requires skin test (commonest comparator in trials)
Hyaluronic acid with dextranomer	Zuidex	Withdrawn in 2009
Porcine dermal implant	Permacol	
Silicone particles	Macroplastique	Recommended by NICE, approved by FDA
Autologous myoblasts	Experimental preparations	Research (not licenced)

FDA, US Food and Drug Administration; NICE, National Institute for Health and Care Excellence.

High-quality long-term evidence for the use of urethral bulking agents as first-line agents is limited. A Cochrane Review in 2017 found only 14 trials of moderate quality which were not suitable for meta-analysis ^23^. Bulking agents were compared with placebo, surgical management or collagen in the trials assessed. In the short term, injectables (Macroplastique) provided a better improvement in continence than conservative treatments ^24^. In two trials which compared surgical treatments with Macroplastique or collagen, surgery resulted in a better objective cure than injectables ^25, 26^. No clear-cut conclusions could be taken from trials comparing different injectable agents.

According to EAU guidelines on urinary incontinence, bulking agents should not be offered to women seeking a permanent cure but may provide short-term improvement in symptoms. Repeat injections may be required ^6^. NICE supports the short-term use of intra-urethral bulking agents but noted that the benefits diminish with time but may be repeated ^7^.

## Burch colposuspension

The Burch colposuspension was initially described in 1961 and involves suspending the anterior vaginal wall to the ileopectinal ligament (Cooper’s ligament). This was the gold-standard surgical approach for many years and can be performed by using the open or laparoscopic approach. Complications include bladder perforation, haemorrhage, *de novo* overactive bladder syndrome and prolapse.

An updated Cochrane Database Review in 2017 of 55 trials involving 5517 women found that the overall continence rate for colposuspension was between 85 and 90% at 1 year after procedure and that 70% of patients remained dry at 5 years. Patient-reported incontinence rates were not significantly different between open and laparoscopic colposuspension, but laparoscopic surgery was found to have a lower rate of complications and a shorter length of stay in hospital ^27^. AUA and EAU guidelines also suggest that open and laparoscopic colposuspension have comparable cure rates.

EAU guidelines suggest offering colposuspension (open or laparoscopic) if a mid-urethral sling (MUS) cannot be considered ^6^. NICE guidelines state that colposuspension, mid-urethral tape or autologous fascial sling can be offered as first-line surgical options but that laparoscopic colposuspension should be offered only by an experienced laparoscopic surgeon working within a multi-disciplinary team ^7^.

## Autologous fascial sling

The autologous fascial pubovaginal sling (PVS) was first popularised by McGuire and Lytton in 1978 and involves harvesting a strip of rectus fascia which is placed suburethrally by using a vaginal incision. In 2007, the Stress Incontinence Surgical Efficacy Trial (SISTEr) reported the results of randomly assigning incontinent women to either PVS or colposuspension. At 24 months, the rate of success (no self-reported SUI, negative stress test and no retreatment) was higher for autologous PVS compared with colposuspension (66% versus 49%), although side effects and need for intervention were more likely ^28^. At 5 years, patient satisfaction remained high (PVS 83% versus 73%) for both procedures, although continence rates had decreased (PVS 30.8%, colposuspension 24.1%) ^29^.

Schimpf *et al*. found autologous PVS to be superior to Burch colposuspension when comparing subjective cure rates (odds ratio [OR] 1.65) and to have a lower incidence of UTI, groin pain and bladder/vaginal perforation ^30^. However, MUS was found to be superior to autologous PVS for subjective cure rates (OR 0.40) and to have a lower incidence of overactive bladder symptoms and retention ^30^.

In 2017, a systematic review of 28 RCTs involving more than 15,000 patients examined comparative data for PVS, colposuspension, tension-free vaginal tape (TVT) and transobturator tape (TOT). Patients undergoing autologous PVS and MUS had similar rates of cure, but MUS had better cure rates than colposuspension. TVT has higher cure rates than TOT though with a higher rate of complications, including bladder perforation, pelvic haematoma, UTIs and voiding symptoms ^31^. Recently, in a small study of the results of transobturator insertion of autologous fascial sling, Linder and Elliott found that 80% of patients were dry at 4 months; however, long-term data are required to validate this approach ^32^.

In summary, PVS has high satisfaction rates at 5 years compared with colposuspension, although popularity waned with the introduction of the MUS, which does not require the additional morbidity of harvesting of fascia. Increasing patient and surgeon concern with mesh-related complications has renewed interest in PVS because of the lack of mesh-type erosion and pain-related complications.

## Mid-urethral synthetic slings

Mid-urethral synthetic sling insertion has become the most common surgical procedure for SUI and largely superseded colposuspension and PVS as a result of the minimally invasive nature and effective cure rate. Ulmsten described the original retropubic tape procedure in 1996: the TVT. This is inserted in a “bottom up” approach through a suburethral incision in the vagina to the anterior abdominal wall posterior to the pubic symphysis. The SPARC (suprapubic arch) sling system is a “top down” retropubic sling with complications similar to those of the TVT but is not commonly performed in the UK. Transobturator tapes were developed in the early 2000s, when Delorme introduced an “outside in” procedure (TOT), and subsequently an “inside out” procedure (TVT-O) was popularised by de Leval ^33^. The Cochrane Review of MUS in 2017 reported increased risk of serious intra-operative complications during TVT compared with TOT, including bladder perforation (0.6% versus 4.5%) and more serious but rare complications such as visceral and vascular injury ( Table 4). Post-operative voiding dysfunction was less frequent after TOT, although the incidence of groin pain was higher (6.4% versus 1.3%) but the incidence of suprapubic pain was lower (0.8% versus 2.9%). The risk of vaginal tape erosion/exposure/extrusion was low in both groups, although the evidence was of low quality ^19^.

**Table 4.** Comparison of transobturator tape and transvaginal tape tapes.

	Transobturator tape	Transvaginal tape	Risk ratio (confidence interval)
Subjective cure 1 year	62–98%	71–97%	0.98 (0.96–1.00)
Subjective cure >5 years	43–72%	51–88%	
Bladder perforation	0.6	4.5	0.13 (0.08–0.2)
Voiding dysfunction	Lower	Increased	0.53 (0.43–0.65)
Groin pain	6.4	1.3	4.12 (2.71–6.27)
Suprapubic pain	0.8	2.9	0.29 (0.11–0.78)
Tape erosion	2.4	2.1	1.13 (0.78–1.65)

Data are from 19.

Fusco *et al*. performed an updated systematic review and meta-analysis of the literature to compare the efficacy and safety of MUS with those of Burch colposuspension and pubovaginal slings ^31^. They found that MUS was significantly superior to Burch colposuspension for overall (OR 0.51) and objective (OR 0.51) cure rates. They had a similar risk of further incontinence surgery and late complications. Retropubic TVT had a higher subjective cure rate (OR 0.83) and objective cure rates (0.82 OR) compared with TVT-O but this was at the cost of higher rates of intra-operative bladder/vaginal perforation, pelvic haematoma and UTI and lower urinary tract symptoms. There were no significant differences in efficacy and safety between “inside out” and “outside in” TVT-O ^31^. There is a risk of intermittent self-catheterisation with both TVT and TVT-O insertion; in a systematic review, Novara *et al*. reported a 1.5% rate following TVT-O and 3.4% following TVT insertion ^19^.

Recently, a single-incision mini-sling placed through a single vaginal incision that can be performed under local anaesthetic as a day case was developed ^34^. However, one type of mini-sling (TVT-Secur,,Ethicon, Somerville, USA) was withdrawn from use because of a higher risk of adverse effects and subsequent data have shown a high failure rate at 3 years compared with retropubic MUS. NICE guidance states that mini-slings should not be used unless special arrangements are in place for clinical governance, consent and research ^7^.

## Mesh safety

In recent years, concerns about complications after the use of synthetic mesh for SUI and pelvic organ prolapse (POP) surgery have been voiced by physicians, regulatory bodies, the media and patient websites. These have identified the risk of complications, including mesh erosion, chronic pain, dyspareunia and infections. In some patients, this has resulted in debilitating symptoms requiring major surgery and in large payouts after successful litigation, particularly in the US.

The Scottish Independent Review of the use, safety and efficacy of transvaginal mesh implants in the treatment of SUI and POP in women was reported in March 2017 and concluded that retropubic mesh tape is a valid option to be offered for SUI ^35^. In addition, Morling *et al*. carried out a cohort study of 16,660 women in Scotland who had undergone a first single incontinence or prolapse procedure with mesh compared with colposuspension ^36^. Mesh procedures were found to have a lower risk of immediate complications (adjusted relative risk of 0.44) and subsequent prolapse surgery. Recent advice from several national bodies in the face of unbalanced reports affecting media, legal and patient perception emphasised that patients should be comprehensively counselled about the risks of mesh implantation, together with conservative options and other surgical options available, before proceeding to mesh implantation, which is supported by good evidence when used in appropriate patients by appropriately trained surgeons.

## Artificial urinary sphincter

The AUS has been used for neurological and recurrent stress incontinence in females for several years but is not in widespread use. Laparoscopic and robotic insertion were first reported in 2015 ^37^ and since then the overall numbers of patients and reporting centres have remained comparatively small. In a case series of 52 women with intrinsic sphincteric deficiency and a median follow-up of 24 months, 77% patients were cured and 22% required revision surgery ^38^. At the end of a study of 26 women with neurogenic incontinence, 58% of patients still had their AUS and 71% of them were continent after a median follow-up of 7.5 years ^39^.

Two systematic reviews of the performance and safety of AMS 800 AUSs (American Medical Systems, Minnetonka, MN, USA) in female patients were published recently ( Table 5) ^20^. The conclusions were based on 12 articles reporting on 886 patients.

**Table 5.** Results and complications of artificial urinary sphincter in women.

Intra-operative complications	Results
Bladder neck injury	0–43.8%
Vaginal injury	0–25%
**Post-operative results**	
Continence	61.1–100%
Follow-up	5–204 months
Erosion	0–22.2%
Explantation	0–45.3%
Mechanical failure	0–44.1%

Data are from 20.

At present, implantation of AUS in female patients is restricted to specialised centres but, despite good success rates, has a high risk of significant morbidity. Well-designed trials with specified indications and long-term follow-up measuring quality-of-life outcomes are required to develop guidelines for AUS implantation in women.

## Conclusions

Options for treatment for refractory SUI in women include autologous fascial slings, mid-urethral tapes and open colposuspension. Although historically colposuspension was the gold standard, now most patients are offered an autologous sling or mid-urethral tape. EAU guidelines recommend offering MUS to women with uncomplicated SUI as the preferred surgical intervention. NICE guidance recommended MUS, open colposuspension or autologous PVS. Surgeons must discuss the risks and benefits of the different procedures and involve the patient in the choice of treatment, and there is currently a moratorium on MUS in the UK. The approach to recurrent stress incontinence after initial surgery depends on reassessment, and an individualised approach including the same options as primary surgery, with the additional option of AUS.

## Male stress incontinence

Male SUI most commonly occurs after treatment of prostate cancer following radical prostatectomy and less commonly following transurethral resection of the prostate (TURP). Post-prostatectomy incontinence (PPI) is likely due to a direct injury to the sphincter or damage to adjacent nerves and soft tissue, whereas post-TURP incontinence is more likely to be due to pre-existing abnormal bladder function. The incidence of male post-prostatectomy SUI ranges from 4 to 74% ^40^. SUI may improve within the first 12 months after surgery, and patients should undergo a trial of pelvic floor muscle training. When conservative management fails, the AUS is considered the gold-standard treatment. Recently, alternatives to the AUS have been developed, such as the male sling and adjustable continence devices in an attempt to make treatment simpler and avoid the mechanical failure risks of the AUS.

### Artificial urinary sphincter

The AMS 721 was first implanted by Scott, Bradley and Timm in 1972. It consists of three components: a control pump, inflatable cuff and pressure-regulating balloon. The AMS 800 has been the most commonly used device since 1983 and its basic design has largely remained unchanged apart from the introduction of an antibiotic coating, sutureless connectors and smaller cuff sizes ^41^. The ZSI 375 device (Zephyr Surgical Implants, Geneva, Switzerland) was introduced in 2007 and is a two-part device consisting of a cuff and pump which can be adjusted percutaneously post-operatively ^40^.

A systematic review by Van der Aa *et al*. found that social continence rates (<1 pad per day) ranged between 61 and 10% in studies with a mean follow-up of more than 24 months and that 43.5% of patients were dry ^42^. Infection rates were 8.5% (3.3–27.8%) and mechanical failure occurred in 6.2% (2–13.8%). Follow-up of patients at more than 10 years has shown overall continence rates of between 59 and 91% ^42^. Yafi *et al*. reported that 79% of patients were dry or had improved continence rates with a 90% increase in quality-of-life scores ^43^.

### Male slings

Male slings were introduced in 1958 to treat PPI. A wide variety of devices are available; these include bone-anchored, transobturator, adjustable and the newer quadratic sling. Bone-anchored male slings (InVance, American Medical Systems) are anchored to the inferior rami of the pubic bone and provide direct compression of the bulbar urethra with reported continence rates of 13 to 66%. The most commonly reported complication was infection (3–15%), a potentially serious complication that ultimately led to withdrawal of InVance from the US market ^40^.

AdVance (American Medical Systems) is a transobturator sling device whose proposed mechanism of action is proximal urethral relocation, although some believe that compression of the bulbar urethra may occur since some patients experience post-operative retention. Rehder *et al*. reported that 76.8% of 156 patients who used the AdVance device were cured or improved at 3 years ^44^. Complications include urinary retention (2.7–15.1%) and perineal pain (4–17%) ^44^. In 2010, the modified AdVanceXP was introduced, and Bauer *et al*. published 36-month follow-up data in a prospective multi-centre study showing a 66% cure rate and 22.5% improvement rate ^45^. There were no intra-operative or long-term complications and no explantations ^45^. Critical success factors for the AdVance sling are good mobility of the sphincter area, and compromising factors to success include radiotherapy, bulking agents, prior TURP and urethral fibrosis.

Adjustable slings allow modification of sling tension post-operatively. The Argus (Promedon SA, Córdoba, Argentina) and Remeex (Neomedic International, Barcelona, Spain) devices provide compression to the bulbar urethra with retropubic traction sutures. Hübner *et al*. showed a success rate of 79% with a median follow-up of 25 months with the Argus device; 39% of patients required adjustment ^46^. The Remeex device has reported success rates of up to 65% at 32 months ^47^. Complications include infection (5–7%), erosion (3–13%) and explantation in 12 to 35%.

The Adjustable Transobturator Male System (ATOMS) (A.M.I., Feldkirch, Austria) is an adjustable device consisting of a urethral pad with mesh on either side, a titanium port for adjustments and a silicon connection. Friedl *et al*. conducted a retrospective multi-centre study of 287 men who used the ATOMS device with a median follow-up of 31 months ^48^. The overall success rate was 90%, and 64% of patients were completely dry. Twenty percent of devices required explantation due to titanium intolerance or leak/dysfunction, and the rate of urinary retention was 3% ^48^.

Virtue (Coloplast, Minneapolis, MN, USA) is a relatively novel sling device with a hybrid design (two transobturator and two pre-pubic arms). The 12 month follow-up outcomes have shown subjective and objective success rates of 70.9% and 79.2% respectively and no severe adverse events or prolonged retention ^49^. A summary of a comparison of male slings is provided in Table 6.

**Table 6.** Results of male sling surgery for stress incontinence.

Sling type	Introduced	Approach	Cure/Dry percentage	Explantation percentage
Adjustable				
Argus T	2006	Transobturator	60	15–9
Reemex	2004	Suprapubic	65	12
ATOMS	2009	Transobtorator	60	20–30
Non-adjustable				
Advance	2007	Transobturator	35–66	1
Virtue	2009	Transobturator	43–79	0

Data are from 21. ATOMS, Adjustable Transobturator Male System.

In summary, male slings are a less invasive treatment of PPI compared with AUS, which is attractive to patients as mechanical manipulation of a pump is not required when wanting to void. Most clinicians would use male slings in those with mild to moderate incontinence and adequate residual sphincter function. There is no accepted definition of when to perform a sling or AUS. The results of the Male synthetic sling versus Artificial urinary Sphincter Trial: Evaluation by Randomised control (MASTER) trial, where patients are randomly assigned to either AUS or male sling, will inform on the relative indications for each treatment.

### Artificial urinary sphincter versus male slings

Crivellaro *et al*. carried out a systematic review of PPI surgical treatments in 2015 ^50^. The authors included articles with more than 12 months of follow-up and defined a successful outcome as the use of zero or one pad per day. They found the AUS to be more efficacious than all sling types (65.7% success AUS, 48.2% InVance and 48.8% AdVance); however, AUS had a higher overall rate of post-operative complications (19.4% versus 7.4% InVance; 12.3% AdVance). However, they also found that the overall quality of studies was poor, and although the AUS had a higher complication rate, this may be due to the longer follow-up ^50^.

## Conclusions

AUS is the gold-standard surgical treatment for male SUI, but the use of male sling procedures is becoming more prevalent. Both procedures have been shown to be safe and efficacious; however, there is a lack of good-quality evidence or long-term outcomes or both. Kumar *et al*. have shown that 75% of patients with moderate SUI prefer a sling over AUS, even against surgeon advice ^51^. The MASTER trial is a multi-centre RCT that is still recruiting. Patients are randomly assigned to either AUS or male sling with outcomes of patient-reported incontinence at 12 months and cost-effectiveness (measured in quality-adjusted life-years at 24 months). The results of this trial should help guide decision making for both patients and clinicians.

## Abbreviations

ATOMS, Adjustable Transobturator Male System; AUA, American Association of Urology; AUS, artificial urinary sphincter; EAU, European Association of Urology; MASTER, Male synthetic sling versus Artificial urinary Sphincter Trial: Evaluation by Randomised control; MUS, mid-urethral sling; NICE, National Institute for Health and Care Excellence; OAB, overactive bladder; oBTXA, onabotulinum toxin A; OR, odds ratio; POP, pelvic organ prolapse; PPI, post-prostatectomy incontinence; PTNS, percutaneous nerve stimulation; PVS, pubovaginal sling; RCT, randomised controlled trial; ROSETTA, Refractory Overactive Bladder: Sacral NEuromodulation vs. BoTulinum Toxin Assessment; SNM, sacral neuromodulation; SNS, sacral nerve stimulation; SUI, stress urinary incontinence; TOT, transobturator tape; TURP, transurethral resection of the prostate; TVT, transvaginal tape; TVT-O, transvaginal tension-free vaginal tape-obturator; UTI, urinary tract infection; UUI, urge urinary incontinence
